# Prevalence of Pathogenic and Likely Pathogenic Variants Associated with Cardiovascular Diseases in Russian Adults and Long-Living Individuals

**DOI:** 10.3390/genes16101228

**Published:** 2025-10-17

**Authors:** Irina Dzhumaniiazova, Elena Zelenova, Veronika Daniel, Mariia Gusakova, Dariia Kashtanova, Mikhail Ivanov, Olga Blinova, Vladimir Yudin, Lorena Matkava, Sergey Mitrofanov, Alexandra Nekrasova, Ekaterina Petriaikina, Marina Erokhina, Aleksey Ivashechkin, Ekaterina Maralova, Olesya Marchenko, Valentina Maksyutina, Valentin Makarov, Anton Keskinov, Sergey Kraevoy, Sergey Yudin

**Affiliations:** Centre for Strategic Planning of the Federal Medical and Biological Agency, 10/1, Pogodinskaya Str., 119121 Moscow, Russia

**Keywords:** genetic risk, pathogenic and likely pathogenic variants, variants of uncertain significance, cardiovascular diseases, whole-genome sequencing, ACMG, InterVar, variant interpretation

## Abstract

**Background:** Cardiovascular diseases remain a leading cause of death worldwide, yet the prevalence of pathogenic and likely pathogenic genetic variants associated with them is still underassessed in some populations. This study aimed to assess the frequency and geographic distribution of such variants within a representative sample of the Russian population. Additionally, it explored potential links between genotype and phenotype in a cohort of long-lived adults. **Methods:** We analyzed whole-genome sequencing data from 75,144 adults and 2,872 individuals aged 90 and older. Variants within 37 ACMG v3.1 genes were examined using InterVar, focusing on nonsynonymous variants and indels across exons and splicing sites. Variants were grouped based on ClinVar (as of 24 April 2023) annotations, with most subjected to manual review to confirm their significance. **Results:** Among the adult participants, 3,817 (5.1%) carried at least one of the variants under consideration. Of these, 141 (0.19%) carried pathogenic, 580 (0.77%) likely pathogenic, and 3,127 (4.16%) variants of uncertain significance. Variants not registered in ClinVar were found in 1,782 individuals (2.37%). Notably, one participant with cardiomyopathy carried a heterozygous TTN variant. In the long-lived cohort, 15 variants were classified as pathogenic or likely pathogenic, alongside 72 uncertain variants; overall, 19 individuals (0.66%) carried pathogenic or likely pathogenic variants. No significant difference was observed in variant frequency between the adult and long-lived groups. **Conclusions:** This study provided essential insights into the prevalence and geographic distribution of cardiovascular disease-related variants in Russia, laying the foundation for targeted genetic screening disease prevention strategies within this population.

## 1. Introduction

Population studies are essential for assessing the prevalence of pathogenic and likely pathogenic variants within different ethnic groups. They aid in identifying populations at increased risk and improving the accuracy of disease risk assessment, while their results guide targeted prevention strategies [1]. Additionally, population studies facilitate the discovery of novel variants, clarify genotype-phenotype relationships, and enhance our understanding of genetic diversity, ultimately advancing personalized medicine and public health [2].

Having comprehensive population data from all countries further enables the identification of variants unique to specific groups, enhances the precision of disease risk predictions, and ensures that medical advances benefit everyone [3]. Without inclusive global representation, certain populations risk being overlooked or misdiagnosed, which can contribute to disparities in healthcare access and outcomes [4]. Ultimately, collecting diverse population data promotes more equitable, effective, and tailored health interventions worldwide, fostering a more comprehensive and inclusive approach to genomic research and healthcare [5].

The Russian population is among the most genetically diverse populations in the world, reflecting the country’s vast geographic expanse and complex history of migration and cultural exchange [6]. This diversity is characterized by a multitude of ethnic groups, each with distinct genetic backgrounds [7].

This diversity poses a critical challenge in delivering precise and effective diagnostic and treatment efforts. The lack of data on the prevalence of variants associated with various diseases, particularly with cardiovascular diseases (CVDs), hampers the understanding of how specific genetic factors contribute to disease risk and impedes the identification of genetic variants most relevant for particular groups, limiting the ability to develop targeted prevention and treatment strategies [8].

Whole-genome sequencing (WGS) is essential in population studies, as it offers a complete view of genetic variation across the entire genome, including rare and structural variants [2]. This comprehensive data facilitates in-depth analysis of genetic diversity, population structure, and disease susceptibility, thereby advancing personalized medicine [9].

More research has focused on population genetics in Russia, highlighting the rich diversity of its ethnic groups [10,11,12]. Additionally, genome-wide association studies (GWAS) are gaining momentum as they seek to identify genetic factors underlying complex diseases [12,13,14,15].

A critical application of WGS in both research and clinical contexts is its role in the interpretation of genetic variants, guided by the standards established by the American College of Medical Genetics and Genomics (ACMG). The ACMG criteria provide a framework to classify variants into categories such as pathogenic, likely pathogenic, uncertain significance, likely benign, or benign. These classifications rely on multiple lines of evidence, including population frequency, computational predictions, functional studies, and case reports.

To effectively implement these standards and streamline the classification process, computational tools like InterVar have been developed. InterVar is designed to automate the application of the ACMG/AMP guidelines, providing a systematic and consistent framework for variant classification. This integration ensures that variants are evaluated based on standardized criteria, which reduces subjective variability and enhances reproducibility across different laboratories and studies [16].

Given that CVDs are the leading cause of mortality worldwide among adults, exploring genetic predispositions to these life-threatening conditions is a vital application of WGS. This study had two main objectives: (1) to assess the prevalence of pathogenic variants linked to disease development across major federal districts of Russia, identifying region-specific genetic risks that may necessitate targeted clinical interventions, and (2) to compare the frequency of these variants among residents of Moscow and its surrounding regions with those found in long-lived individuals from the same area. To ensure accurate and consistent classification of these variants, we utilized computational tools like InterVar, which automate the application of ACMG guidelines, thereby enhancing the reliability and reproducibility of our findings. By examining these variations, we aimed to better understand the genetic factors influencing cardiovascular health and longevity within the diverse Russian population.

## 2. Methods

### 2.1. Participants

Between 2019 and 2022, the Centre for Strategic Planning and Management of Biomedical Health Risks of the Federal Medical and Biological Agency conducted a large-scale epidemiological study to assess the prevalence of hereditary genetic variants associated with chronic diseases, including cardiovascular conditions, across 79 regions of Russia. Participants were randomly selected to form a representative sample, totaling 75,144 individuals. Participants self-reported their ethnicity, and those with diagnosed conditions reported their health status during data collection. Genetic variants of interest were also analyzed in a cohort of long-lived individuals aged 90 and above from Moscow, comprising 2872 participants, as detailed in our prior publication [17]. The study was approved by the ethics committee of the Centre for Strategic Planning and Management of Biomedical Health Risks (excerpts from protocols No. 5, dated 28 December 2020, and No. 2, dated 1 June 2021). All participants provided written informed consent to participate in the study.

The inclusion of long-living individuals in the research and the examination of their genetic traits were approved by the ethics committee of the Separate Structural Unit Russian Gerontology Research and Clinical Centre (RGRCC) of the Federal State Autonomous Educational Institution of Higher Education Pirogov Russian National Research Medical University of the Ministry of Health of the Russian Federation (excerpt from protocol No. 30, 24 December 2019).

### 2.2. Whole-Genome Sequencing

Venous blood samples were collected between 2019 and 2021 in EDTA tubes for subsequent DNA extraction and sequencing. The samples were cryopreserved to ensure their integrity for downstream analyses.

DNA extraction from whole blood samples was performed using the QIAamp DNA Mini Kit (Qiagen, Hilden, Germany). Library preparation for whole-genome sequencing was carried out using the Nextera DNA Flex Kit (Illumina, San Diego, CA, USA). Sequencing was carried out on the Illumina NovaSeq 6000 system with the S4 Reagent Kit (300 cycles) (Illumina, San Diego, CA, USA), generating 150 bp paired-end reads.

Sequencing data in BCL format were demultiplexed using the Illumina bcl2fastq2 v2.20.0.422 software to generate FASTQ files. Sequencing quality was monitored using Illumina Sequencing Analysis Viewer v2.4.7, and read quality control was performed on FASTQ.GZ files using FastQC v0.11.9. The reads were mapped to the GRCh38.d1.vd1 reference genome using the Illumina DRAGEN Bio-IT Platform v07.021.510.3.5.7. The quality of the alignments in the BAM files was evaluated using DRAGEN FastQC v0.11.9, SAMtools v1.13, and mosdepth v0.3.1. Each sample was evaluated for duplicates, unmapped reads, and additional quality metrics. The mean sequencing coverage for all samples was 30x. Small variant calling (up to 50 bp) was performed using Illumina Strelka2 v2.9.10 [18]. Individuals were excluded when identified as a low-quality sample (individual call rate < 0.98), heterozygosity outlier (F ± 0.20), gender mismatch (females: F > 0.2, males: F < 0.2) when comparing phenotypic and genotypic data or being related to another sample (PI_HAT > 0.2). Variants not conforming to the Hardy–Weinberg equilibrium (*p* < 10^−6^), a call rate > 0.98, and multiallelic variants were removed from the study.

To adjust for population heterogeneity, a principal component analysis (PCA) was performed on a dataset of 15,000 SNPs from the Human Core Exome SNP Array (Illumina), with a frequency of less than 1% using Scikit-learn. The consistency of the results was validated through more than 50 simulations, with a variance of less than 5%.

### 2.3. Variant Interpretation

Variants interpretation followed the protocol employed in our previous study [17].

### 2.4. Statistical Analysis

Cluster analysis was carried out using the clustermap function of the Seaborn library in Python v0.11.2. The differences in the frequency of occurrence of single nucleotide variants (SNVs) were compared using a two-proportion z-test, implemented via the proportion z-test function in Statsmodels v0.13.2. The Benjamini–Hochberg procedure was applied to control for the false discovery rate arising from multiple comparisons.

Additional statistical analyses and visualization were carried out using Python (v3.9.12) and its libraries: Numpy (v1.21.5), Pandas (v1.4.2), Seaborn (v0.11.2), and Matplotlib (v3.5.1).

Data in this study are primarily expressed as median with interquartile range, unless otherwise specified.

## 3. Results

### 3.1. Sample Descriptions

The general population sample comprised 75,144 adults from 79 regions of the Russian Federation, including 37,407 women (49.78%; median age = 51 [42; 59]) and 37,737 men (50.22%; median age = 50 [42; 58]) (*n* = 75,144). Self-reported ethnicity was available for 60,751 participants: 50,953 were Russians (83.9%), 1,636 were Tatars (2.7%), 1,173 were Chuvash (1.9%), 1,019 were Ingush (1.7%), 775 were Ukrainians (1.3%), and 666 were Kabardian (1.1%). A total of 7.5% of individuals reported their ethnic background as “other”.

The cohort of long-living individuals included 2,153 women (75.0%; median age = 92 years [91, 94]) and 719 men (25%; median age = 92 years [91, 94]). Supplementary Figure S1 shows the sex and age structure in both study samples. Self-reported ethnicity was available for 2,732 individuals (95.13%): 2,507 were Russian (91.76%), 62 were Ukrainian (2.27%), 51 were Jewish (1.86%), 39 were Tatar (1.43%), 21 were Belarusian (0.77%), 15 were Armenian (0.55%), 5 were Mordovian (0.18%), 4 were Chuvash (0.15%), 3 were Latvian (0.11%), 3 were Georgian (0.11%), 3 were Polish (0.11%), 2 were German (0.07%), 2 were Greek (0.07%), 2 were Bashkir (0.07%), and 2 were Moldavian (0.07%). Eleven individuals (0.42%) reported their ethnic background as “other”.

### 3.2. WGS and Variant Annotation Results

The total number of SNPs per sample in the general population sample (*n* = 75,144) was 3,979,023 [3,960,245; 3,997,021], and 3983,527 [3,967,608; 3,997,031] in the cohort of long-living individuals (*n* = 2,872). The Ti/Tv ratio was 1.966 [1.964; 1.968] and the Het/Hom ratio was 1.679 [1.647; 1.709] in the general population sample and 1.966 [1.964; 1.969] and 1.648 [1.629; 1.678], respectively, in the cohort of long-living individuals. 

Whole-genome sequencing identified 623,490 variants across 37 genes of interest, which were unique to the general population sample and long-living individuals. After initial filtration based on location, type, and frequency in GnomAD (see the Section 2), 10,147 unique variants remained, accompanied with InterVar predictions, in silico interpretations, and ClinVar annotations. As a result of the semi-automated variant review, 58 variants were interpreted as PVs in *MYH7*, *LDLR*, *MYBPC3*, *KCNQ1*, *DSP*, *SCN5A*, *TTN*, *KCNH2*, *COL3A1*, *FLNC*, *PKP2*, *PRKAG2*, *LMNA*, *TPM1*, *APOB*, *BAG3*, and *DES*; 210 variants were interpreted as LPVs in *LDLR*, *KCNQ1*, *MYL2*, *FBN1*, *MYBPC3*, *TRDN*, *MYH7*, *TTN*, *COL3A1*, *SCN5A*, *DSP*, *DSG2*, *PKP2*, *LMNA*, *FLNC*, *KCNH2*, *TPM1*, *CASQ2*, *TMEM43*, *ACTA2*, *MYL3*, *MYH11*, *DSC2*, *DES*, and *TNNC1*; and 1,408 variants were interpreted as VUSs in *SCN5A*, *APOB*, *TTN*, *LDLR*, *DSP*, *COL3A1*, *KCNQ1*, *MYBPC3*, *ACTC1*, *DSG2*, *TGFBR2*, *KCNH2*, *MYH7*, *MYH11*, *LMNA*, *FLNC*, *DSC2*, *TNNT2*, *PRKAG2*, *MYL3*, *MYL2*, *TMEM43*, *TPM1*, *TRDN*, *FBN1*, *CASQ2*, *RYR2*, *TGFBR1*, *ACTA2*, *PKP2*, *SMAD3*, *PCSK9*, *DES*, *RBM20*, *TNNC1*, and *BAG3.* A total of 1016 VUSs were not registered in ClinVar and were interpreted by InterVar as PVs or LPVs. These are further referred to as novel variants. No PVs, LPVs, or VUSs were found in TNNI3. Supplementary Table S1 presents all detected variants and their final interpretations.

**Figure 1:**
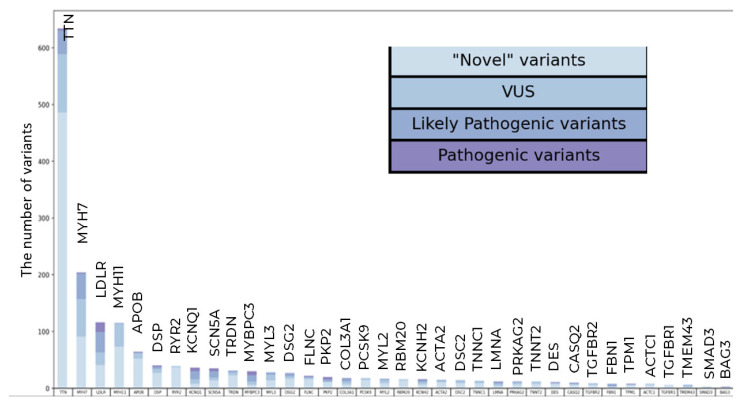
Qualitative and clustering data analysis in the general population sample. The total number of PVs, LPVs, and novel VUSs variants per gene.

The highest number of variants were found in *TTN* (three PVs, 42 LPVs, and 589 VUSs) and *MYH7* (2 PVs, 45 LPVs, and 157 VUSs) (Figure 1).

A total of 3,817 participants (5.1%) carried at least one variant; 141 participants (0.19%) carried PVs and 580 (0.77%) carried LPVs. There were 3,127 (0.77%) carriers of VUSs, including 1,714 VUSs not registered in ClinVar. Two or more variants were detected in 122 participants. One participant carried three novel VUSs in *TTN*: chr2:178564878G>T, chr2:178631070C>A, and chr2:178741687G>T.

**Figure 2:**
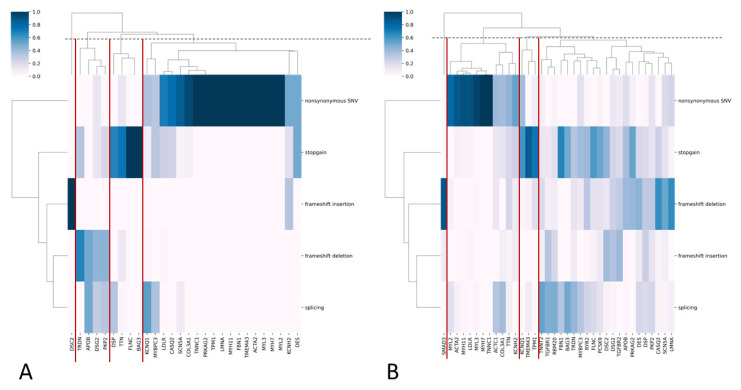
(**A**,**B**) Heatmaps with hierarchical clustering, where the y-axis represents functional variant types and the x-axis represents genes for PVs/LPVs (**A**) and VUS (**B**). The color indicates the frequency of each variant type in each gene. The dendrograms cluster genes with similar variant profiles. Gene clusters are highlighted with red lines.

Most PVs and LPVs were nonsynonymous substitutions, while most VUSs were nonsynonymous and stop-gain substitutions (Figure 2A,B).

The highest frequency of occurrence in healthy participants among all VUSs was demonstrated by rs565663412 in *MYH7* (AF = 0.00145; 218 alleles). The highest frequency of occurrence in healthy participants among all PVs and LPVs was shown by rs730880808 in *MYH7* (AF = 0.000313, 47 alleles), rs137929307 in *LDLR* (AF = 0.00022, 33 alleles “reviewed by expert panel” in ClinVar), and rs762814879 in *KCNQ1* (AF = 0.00022, 33 alleles) (Supplementary Table S2).

Table 1 presents the total number and the frequency of occurrence of PVs, LPVs, and VUSs in each gene.

### 3.3. Frequency Analysis of PVs and LPVs in the Federal Districts of Russia

The frequency of occurrence of PVs, LPVs, and VUSs was calculated for each region (Supplementary Table S3). The median frequency was 0.00434 [0.00157, 0.00584] for PVs and LPVs and 0.0192 [0.0167, 0.0267] for VUSs. Furthermore, allele frequencies were calculated only for PVs and LPVs as variants recommended for reporting by ACMG.

**Figure 3:**
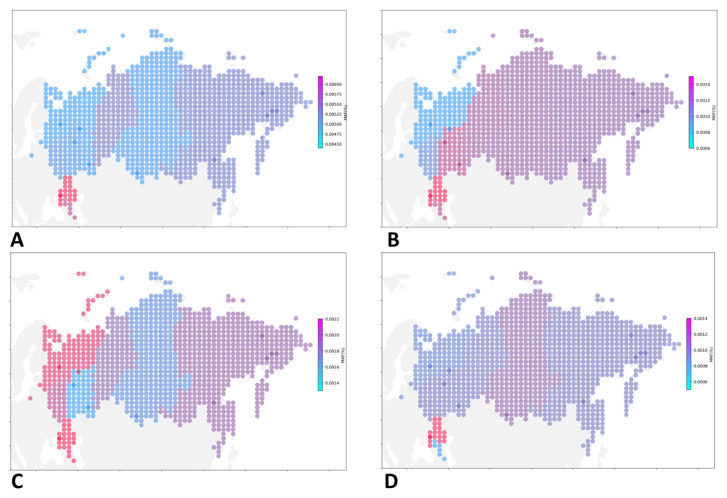
Geographic distribution pf PVS and LPVs associated with cardiovascular diseases. A spatial distribution map illustrating the prevalence of CVD-associated PVs and LPVs in various parts of Russia (**A**). PVs and LPVs associated with familial hypercholesterolemia (**B**), PVs and LPVs associated with hypertrophic cardiomyopathy (**C**), and PVs and LPVs associated with dilated cardiomyopathy (**D**).

To improve the quality of visualization, we used transformed data on the frequency of occurrence of all pathogenic and likely pathogenic variants (Figure 3A) and variants associated with specific CVDs (Figure 3B–D; Supplementary Table S4).

PVs and LPVs in genes associated with familial hypercholesterolemia were most common in the Southern Federal district (AF = 0.00155), North Caucasian Federal district (AF = 0.00125), Volga Federal district (AF = 0.00124); with hypertrophic cardiomyopathy, in the North Caucasian Federal district (AF = 0.00223), Southern Federal district (AF = 0.00222), Northwestern Federal district (AF = 0.00212); with dilated cardiomyopathy, in the Southern Federal district (AF = 0.00145), Siberian Federal district (AF = 0.00096), Central Federal district (AF = 0.00085); with Long QT Syndrome 3 and Brugada syndrome, in the Volga Federal district (0.00052), Southern Federal district (AF = 0.00048), Siberian Federal district (AF = 0.00030); with the Ehlers-Danlos syndrome, in the North Caucasian Federal district (AF = 0.00048), Far Eastern Federal district (0.00010), Ural federal district (AF = 0.00008); with arrhythmogenic right ventricular cardiomyopathy, in the Central Federal district (AF = 0.00023), Northwestern Federal district (AF = 0.00022), Far Eastern Federal district (AF = 0.00021); with long QT1 and QT2, in the Ural Federal district (0.00076), Far Eastern Federal district (AF = 0.00073), and Northwestern Federal district (AF = 0.00053).

### 3.4. PV and LPV Carriers in the General Population Sample

VUSs can rapidly move from one clinical category into another once new data becomes available. Therefore, 3,127 VUS carriers were further examined, including carriers of novel variants. Diagnoses were known for 809 of these participants (25.87%). Carriers with polyetiological diseases were excluded from further analysis. The analysis focused on carriers of CVD-associated genes recommended for reporting by the ACMG, particularly those associated with cardiomyopathies (I42), cardiac arrhythmias and conduction disorders (I49, I45), and pure hypercholesterolemia (E78.0). Several participants with these CVDs carried novel VUSs. Some of these VUSs were located in genes whose functional changes may lead to the aforementioned CVDs (Supplementary Table S5).

### 3.5. CVD-Associated Variants in the Cohort of Long-Living Individuals

Four PVs were detected in *SCN5A*, *KCNQ1*, and *LDLR*; 11 LPVs were detected in *DSP*, *MYH7*, *MYBPC3*, *TTN*, *MYL2*, *SCN5A*, and *TNNC1*; and 71 VUSs were detected in *KCNQ1*, *ACTC1*, *APOB*, *DSC2*, *TTN*, *MYH7*, *MYL2*, *KCNH2*, *LDLR*, *MYL3*, *MYH11*, DSG2, *TMEM43*, *PKP2*, *LMNA*, *RYR2*, *TRDN*, *RBM20*, *TNNC1*, and *DES*. Forty-two of these VUSs were novel variants. Supplementary Table S6 presents all variants and the results of their review. No PVs were found in *FBN1*, *TGFBR1*, *TGFBR2*, *SMAD3*, *ACTA2*, *CASQ2*, *TNNT2*, *FLNC*, *BAG3*, *COL3A1*, *PCSK9*, *TNNI3*, *TPM1*, or *PRKAG2*.

**Figure 4:**
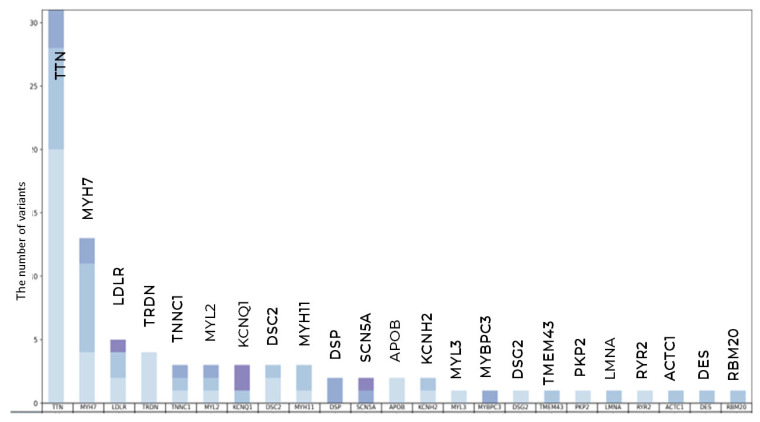
Qualitative and clustering data analysis in long-living individuals. The total number of PVs, LPVs, and novel VUSs variants per gene.

The highest number of variants was found in *TTN*—0 PVs, 3 LPVs, and 28 VUSs—and in *MYH7*—0 PVs, 2 LPVs, and 11 VUSs (Figure 4).

**Figure 5:**
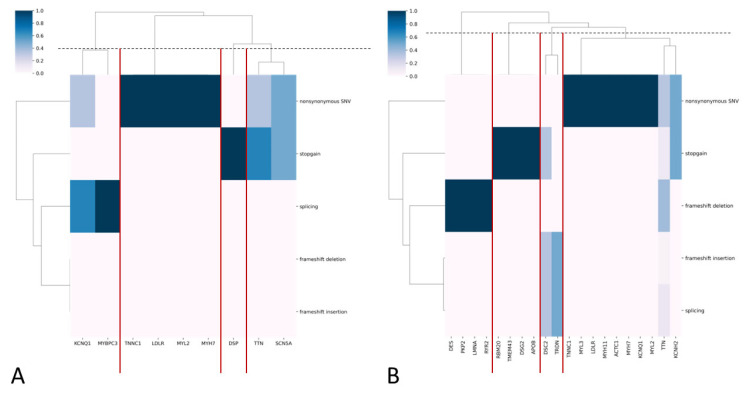
(**A**,**B**) Heatmaps with hierarchical clustering, where the y-axis represents functional variant types, and the x-axis represents genes for PVs/LPVs (**A**) and VUS (**B**). The color indicates the frequency of each variant type in each gene. The dendrograms cluster genes with similar variant profiles. Gene clusters are highlighted with red lines.

Overall, 104 long-living individuals (3.62%) carried at least one variant: 6 (0.21%) carried PVs, 13 (0.45%) carried LPVs, and 87 carried VUSs, including 47 novel VUSs. Five long-living participants simultaneously carried two VUSs. PVs and LPVs were predominantly nonsynonymous substitutions, while VUSs were mainly nonsynonymous substitutions, frameshift deletions, and stop-gain mutations (Figure 5A,B).

Supplementary Table S7 presents the frequency of occurrence of each variant. The rs781363456 variant in *TTN* demonstrated the highest frequency of occurrence (VUS; AF = 0.000870; 5 alleles). The rs730880806 variant in *MYH7* (AF = 0.00035; 3 alleles). rs2071674449 in MYL2 (AF = 0.00035; 2 alleles) and the rs762814879 variant in *KCNQ1* (AF = 0.00035; 2 alleles) showed the highest frequency of occurrence among all PVs and LPVs.

Some gene variants were found both in the general population sample and the cohort of long-living individuals. A comparison of the frequencies of variants common to both the general population sample and the cohort of long-living individuals was conducted only for residents of Moscow and the Moscow region, as the vast majority of older adults (2,810 out of 2,872) originated from these areas. Of the 86 variants reported, 41 were found only in the general population sub-sample: two PVs, five LPVs, and 34 VUSs. Supplementary Table S8 presents all common variants. Table 2 presents common PVs and LPVs. The median frequency of occurrence of common PVs was 0.000174 [0.000174; 0.000261] in the cohort of long-living individuals and 0.000160 [0.000093; 0.000213] in the general population sub-sample.

**Figure 6:**
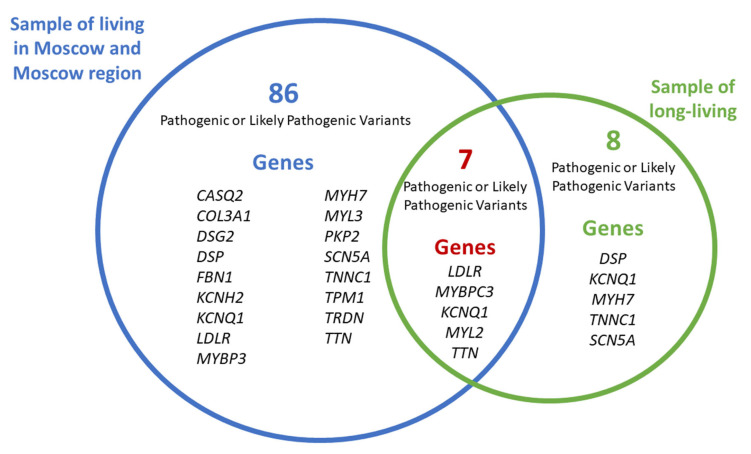
Common and unique PVs and LPVs in the general population sample and the cohort of long-living individuals from Moscow and the Moscow region.

Figure 6 shows the total number of PVs and LPVs in the general population sub-sample and the cohort of long-living individuals from Moscow and the Moscow region, as well as seven variants common to both groups.

The following PVs and LPVs were unique to the cohort of long-living individuals from Moscow and the Moscow region and were not detected in the general population sub-sample:

SCN5A (SCN5A:NM_000335:exon5:c.C611T:p.A204V),
DSP (DSP:NM_001008844:exon23:c.C3337T:p.R1113X, DSP:NM_001008844:exon24:c.C6232T:p.Q2078X),
KCNQ1 (KCNQ1:NM_000218:exon3:c.G535A:p.G179S),
MYH7 (MYH7:NM_000257:exon31:c.C4258T:p.R1420W),
TNNC1 (TNNC1:NM_003280:exon4:c.G248A:p.R83Q).

## 4. Discussion

In this study, we assessed the prevalence of pathogenic (PVs) and likely pathogenic variants (LPVs) within the Russian Federation, with a specific focus on the general population and long-living individuals from Moscow and the Moscow region. The study had two primary objectives: (1) to determine the regional distribution of pathogenic variants implicated in cardiovascular diseases (CVDs) across major federal districts, thereby identifying region-specific diseases that warrant clinical consideration, and (2) to compare the frequency of these variants between the general population sample and the cohort of long-living individuals.

We focused on 37 genes recommended for reporting by the ACMG v3.1 [19] as genes associated with various CVDs, such as aortopathies, cardiomyopathies, and arrhythmias. Given that most of these variants exhibit autosomal dominant inheritance, the presence of a single pathogenic allele can predispose individuals to disease development. To streamline the analysis while addressing limitations inherent to public databases and automated interpretations, we employed a semi-automated algorithm. This approach integrated InterVar interpretations, ClinVar annotation, and available data.

Our analysis highlights the gap in the representation of non-Western European ancestry in public genetic databases; a majority of variants we identified were absent from resources like GnomAD (Supplementary Tables S1 and S6). Yet, the overall carrier frequency of pathogenic and likely pathogenic variants was consistent with other population studies at slightly below 1% [20]. While valuable existing studies of the Russian population have focused on genome-wide associations and population history, the prevalence of rare and potentially pathogenic variants remains underassessed. The RuSeq database [11] represents a significant step forward, though its primary focus remains population genetics, reporting an increased frequency of previously known pathogenic variants. Our study aimed to address this specific need by evaluating the prevalence of variants whose population frequency had not been previously established.

Our analysis prioritized pathogenic (PVs) and likely pathogenic variants (LPVs) for their direct clinical implications. A key metric in this assessment is the maximal pathogenic allele frequency (MPAF), which defines the upper allele frequency threshold for a disease phenotype within a population. By comparing observed allele frequencies against established MPAF values, we can refine the evaluation of a variant’s pathogenicity and rarity in healthy individuals. We assessed the overall frequency of PVs, LPVs, and variants of uncertain significance (VUSs) in each gene (Table 1). The highest frequency of PVs and LPVs was observed in *MYH7* (0.00113), which was below the estimated MPAF for this gene [21]. Given the variable penetrance of *MYH7* variants, they may be present in healthy older individuals [22]. The frequency of PVs and LPVs in other genes was also lower than their estimated MPAF [21]. Notably, no variants recommended for reporting by the ACMG for secondary clinical findings were detected in *TGFBR1, TGFBR2, SMAD3, RYR2, TNNT2, RBM20, PCSK9, ACTC1,* or *TNNI3* in the general population sample. Given that pathogenic variants in these genes lead to the development of clinically significant phenotypes, their presence in a healthy older adult is highly unlikely. Additionally, the absence of PVs in most of these genes among long-living individuals further reinforces the association between PVs and LPVs and severe clinical manifestations.

In the general population sample, we categorized genes into four groups based on the predominant functional types of PVs and LPVs (Figure 2A). Nonsynonymous substitutions and stop-gain variants were the most common classes across genes. This is likely attributable to the established overrepresentation of these variant types in ClinVar (see Section 2).

Our analysis revealed a frequency of PVs and LPVs associated with hypercholesterolemia and cardiomyopathies across different genes (Supplementary Table S4), underscoring a need for targeted screening and prevention strategies. The highest overall frequency of PVs and LPVs was observed in the Southern Federal District (AF = 0.00619; *n* = 5,137), the North Caucasian Federal District (AF = 0.00561; *n* = 5,612), and the Ural Federal District (AF = 0.00498; *n* = 6,621), highlighting these regions as priorities for focused CVD screening.

In the cohort of long-living individuals, no reportable variants were found in *FBN1* (AF in the general population sample = 0.0000399), *TGFBR1* (AF = 0), *TGFBR2* (AF = 0), *SMAD3* (AF = 0), *TNNI3* (AF = 0), *ACTA2* (AF = 0.0000200), *CASQ2* (AF = 0.0000266), *TNNT2* (AF = 0), *FLNC* (AF = 0.0000266), *BAG3* (AF = 0.0000067), *COL3A1* (AF = 0.000107), *PCSK9* (AF = 0), *TPM1* (AF = 0.0000133), and *PRKAG2* (AF = 0.0000067). The absence of these variants in long-living individuals, contrasted with their low but non-zero frequency in the general population sample, supports the hypothesis that pathogenic variants in these highly conserved genes likely have a negative impact on longevity. It is also noteworthy that these are highly conserved genes. It should be noted, however, that the sample size (*n* = 2,872) limits the power to detect extremely rare variants, and thus these findings should be interpreted as suggestive rather than conclusive.

Our comparison of genetic profiles of the general population sample and long-living individuals offers valuable insights into genetic factors that may promote longevity. For this comparison, long-living individuals were recruited from Moscow and the Moscow region, while the general population sample comprised adults from across the Russian Federation. We acknowledge that differences in genetic ancestry and population structure between these groups could potentially influence the results of such a comparison. However, this approach was selected to provide a broader, more robust epidemiological perspective on the national distribution of genetic variants. We identified eight PVs and LPVs that were present in both cohorts with similar frequencies. This finding indicates that carrying these variants is compatible with longevity. The variants common to both groups included the following:

SCN5A:NM_000335:exon12:c.C1603T:p.R535X,
SCN5A:NM_000335:exon5:c.C611T:p.A204V,
DSP:NM_001008844:exon23:c.C3337T:p.R1113X,
DSP:NM_001008844:exon24:c.C6232T:p.Q2078X,
KCNQ1:NM_000218:exon3:c.G535A:p.G179S,
MYH7:NM_000257:exon31:c.C4258T:p.R1420W,
and TNNC1:NM_003280:exon4:c.G248A:p.R83Q.

The presence of these specific variants in the long-living cohort suggests they may not adversely impact survival to advanced age, potentially due to lower penetrance or modification by other genetic, environmental, or lifestyle factors. However, further functional and cohort studies are essential to definitively characterize their clinical impact and role in longevity.

We also report findings on VUSs suggestive of potential pathogenicity. While direct clinical evidence is currently limited, the predominance of loss-of-function mechanisms, including frameshift deletions, frameshift insertions, splicing, and stop-gains (Figure 2B), consistent with InterVar’s application of the PVS1 (very strong pathogenicity) criterion, warrants their prioritization for future clinical and functional validation. Notably, the majority of these VUSs (1,064 out of 1,408) are novel, lacking entries in ClinVar, yet were assigned a pathogenic prediction by InterVar, which again may arise from InterVar’s application of the above criterion. Nevertheless, this finding suggests the existence of a set of not previously cataloged novel variants with potential clinical implications. We therefore posit that reporting these variants provides a valuable resource for the clinical and research communities, enabling proactive assessment of their pathogenicity as new evidence emerges. These variants and their corresponding genes exhibited a greater functional diversity than PVs and LPVs, which was also evident in the cohort of long-living individuals (Figure 5A,B), where most PVs and LPVs were nonsynonymous substitutions or stop-gains, while VUSs were mostly frameshift deletions, insertions, and splicing events.

## 5. Conclusions

This study underscores a critical lack of representation of non-Western European ancestry, including Russian populations, in global genetic databases like gnomAD. A significant portion of the variants identified were absent from these resources, highlighting an inherent bias that can lead to missed diagnoses and inaccurate variant interpretation in underrepresented groups. This finding highlights the urgent need to diversify genetic databases to make precision medicine fair and accurate for everyone.

Our findings on PVs and LPVs provide crucial insights for medical genetics. We established the baseline carrier frequencies for these variants in the Russian population, which enables improved genetic risk assessment and informs the development of population-specific screening programs. Importantly, we identified several such variants that are present in long-living individuals, demonstrating that some deleterious variants can be compatible with exceptional longevity, likely due to incomplete penetrance or modifying factors. This finding refines our understanding of genetic pathogenicity and has direct implications for more accurate genetic counseling. Furthermore, our identification of geographic regions with higher variant frequencies offers valuable guidance for targeting clinical resources and preventive health initiatives where they are most needed.

In summary, this study provides a foundational assessment of the carrier burden of rare genetic variants in the Russian population, identifies genetic factors that may influence human longevity, and highlights the necessity of population-specific studies to overcome the biases of current global databases, thereby contributing to more inclusive and accurate genetic medicine.

## 6. Limitations

1. Our variant assessment relied on the InterVar software (https://wintervar.wglab.org/, accessed on 27 July 2021), which provides a standardized, evidence-based classification. It is important to note that this automated approach does not incorporate patient-specific data, such as detailed family health history, or functional evidence from in vitro or in vivo studies.
2. The InterVar framework is optimized for classifying highly penetrant variants causative for congenital or early-onset Mendelian disorders. However, the pathogenicity of variants associated with common cardiovascular diseases (CVDs) is often modulated by significantly reduced penetrance and polygenic influences. For instance, pathogenic variants in genes associated with dilated cardiomyopathy have a penetrance of 17% [23], whereas the pathogenic variant in the LDLR gene (Ser177Leu) associated with familial hypercholesterolemia has a penetrance of 67% [24]. This reduced penetrance is a known challenge for automated classification systems and is a critical consideration when interpreting the potential disease risk associated with identified variants in our cohort.
3. In this study, we did not correct for genetic ancestry, whether calculated or self-reported. Our main goal was to establish the prevalence and geographic distribution of genetic variants across Russia. This focus on geography rather than ancestry produces results that are more clinically actionable and captures the real-world genetic makeup of regional populations, thereby offering practical insights guiding diagnostic and screening efforts specific to these areas.
4. The size of the cohort of long-living individuals was smaller than our general population sample, which inherently reduces the power to detect very low-frequency variants in this group. Despite this limitation, we have included the findings from this cohort, as it represents a unique resource for identifying genetic factors associated with healthspan and longevity. The absence of certain pathogenic variants in this group may yield valuable hypotheses for future research into protective genetic mechanisms.

These limitations should be considered when generalizing our findings, and further studies with additional ancestry analysis will be essential to validate and expand our results.

## Figures and Tables

**Table 1:** Total number and frequency of occurrence of CVD-associated PVs, LPVs, and VUSs in the general Russian population sample.

Gene	Total Number PV and LPV Alleles	Frequency of Occurrence of PVs and LPVs	Total Number of VUS Alleles	Frequency of Occurrence of VUSs
*ACTA2*	3	0.0000120	16	0.000106
*ACTC1*	0	0	15	0.0000998
*APOB*	2	0.0000133	133	0.000885
*BAG3*	1	0.00000665	2	0.0000133
*CASQ2*	4	0.0000266	19	0.000126
*COL3A1*	16	0.000106	13	0.0000865
*DES*	2	0.0000133	18	0.000120
*DSC2*	1	0.00000665	21	0.000140
*DSG2*	7	0.0000466	38	0.000253
*DSP*	6	0.0000400	44	0.000293
*FBN1*	6	0.0000400	3	0.0000200
*FLNC*	4	0.0000266	19	0.000126
*KCNH2*	6	0.0000399	19	0.000126
*KCNQ1*	62	0.000413	40	0.000266
*LDLR*	147	0.000978	89	0.000592
*LMNA*	4	0.0000266	25	0.000166
*MYBPC3*	74	0.000492	18	0.000120
*MYH11*	1	0.00000665	257	0.00171
*MYH7*	169	0.00112	550	0.00366
*MYL2*	17	0.000113	18	0.000120
*MYL3*	15	0.0000998	66	0.000439
*PCSK9*	0	0	25	0.000166
*PKP2*	9	0.0000600	13	0.0000865
*PRKAG2*	1	0.00000665	17	0.000113
*RBM20*	0	0	23	0.000153
*RYR2*	0	0	42	0.000279
*SCN5A*	40	0.000266	25	0.000166
*SMAD3*	0	0	9	0.0000599
*TGFBR1*	0	0	15	0.0000998
*TGFBR2*	0	0	10	0.0000665
*TMEM43*	1	0.00000665	22	0.000146
*TNNC1*	2	0.0000133	26	0.000173
*TNNT2*	0	0	15	0.0000998
*TPM1*	2	0.0000133	11	0.0000732
*TRDN*	3	0.0000200	93	0.000619
*TTN*	117	0.000779	1449	0.00964

**Table 2:** Comparison of the frequency of occurrence of PVs and LPVs found both in the general sample and cohort of long-living individuals from Moscow and the Moscow region.

Chromosome	Positions	Reference Allele	Alternative Allele	Gen	Annotation	Number of Alleles in the General Sample	Frequency of Occurrence in the General Sample	Number of Alleles in the Sub-Sample	Frequency of Occurrence in the Sub-Sample	*p*-Value (Z-Test). Correction for Multiple Testing: FDR (Bonferroni.-Hochberg)
2	178,531,025	C	T	*TTN*	Likely pathogenic	3	0.0000798	1	0.000174	0.575
2	178,665,777	G	A	*TTN*	Likely pathogenic	1	0.0000266	1	0.000174	0.177
2	178,739,537	G	A	*TTN*	Likely pathogenic	8	0.000213	1	0.000174	0.876
11	2,528,019	G	A	*KCNQ1*	Pathogenic	8	0.000213	2	0.000348	0.601
11	47,351,507	T	C	*MYBPC3*	Likely pathogenic	4	0.000107	1	0.000174	0.690
12	110,914,254	A	G	*MYL2*	Likely pathogenic	6	0.000160	2	0.000348	0.390
19	11,116,928	G	A	*LDLR*	Pathogenic	8	0.000213	1	0.000174	0.876

## Data Availability

The data supporting the findings of this study are available from the corresponding author upon request.

## Supplementary Materials

The following supporting information can be downloaded at https://www.mdpi.com/article/10.3390/genes16101228/s1: Table S1: PVs, LPVs, and VUSs in the general population sample; Table S2: Allele frequency of PVs, LPVs, and VUSs in the general population sample; Table S3: Allele frequency of PVs, LPVs, and VUSs in the Federal districts of Russia; Table S4: Allele frequency of PVs, LPVs, and VUSs associated CVDs in the Federal districts of Russia; Table S5: Phenotypes of VUS carriers; Table S6: PVs, LPVs, and VUSs in the cohort of long-living individuals; Table S7: Allele frequency of PVs, LPVs, and VUSs in the cohort of long-living individuals; Table S8: Comparison of variants common to the general population sample and the cohort of long-living individuals; Figure S1: Sex and age distributions within the study cohorts. (A) Sex and age distributions of the general population sample. (B) Sex and age distributions of the cohort of long-living individuals.
