# Supplementary figures and images for: Prevalence of Pathogenic and Likely Pathogenic Variants Associated with Cardiovascular Diseases in Russian Adults and Long-Living Individuals
Source: Genes (Basel). 2025 Oct 17;16(10):1228. doi: 10.3390/genes16101228 (PMC12562750; doi:10.3390/genes16101228)

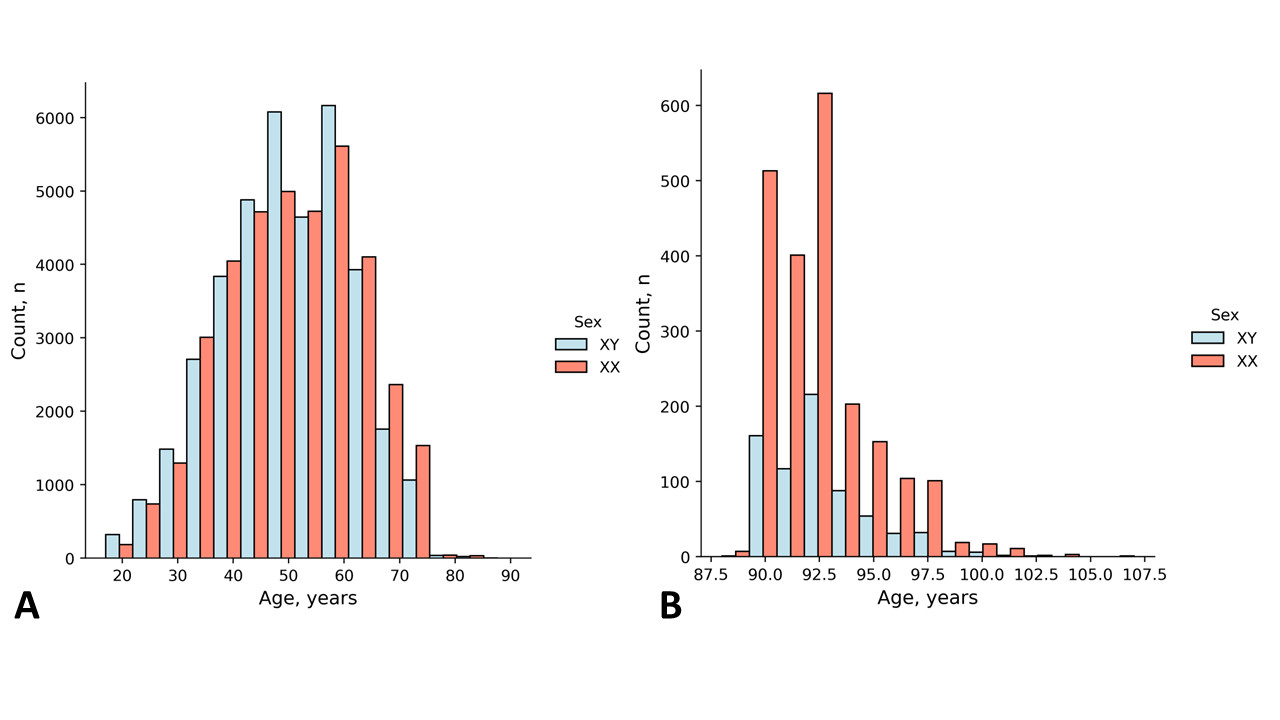

Supplement: Supplementary file 1 [file genes-16-01228-s001.zip › Fig S1.jpg]
